# Informing policy makers on the efficiency of population level tobacco control interventions in Asia: A systematic review of model-based economic evaluations

**DOI:** 10.7189/jogh.10.020437

**Published:** 2020-12

**Authors:** Ariuntuya Tuvdendorj, Yihui Du, Grigory Sidorenkov, Erik Buskens, Geertruida H de Bock, Talitha Feenstra

**Affiliations:** 1 University of Groningen, University Medical Center Groningen, Department of Epidemiology, Groningen, the Netherlands; 2 Mongolian National University of Medical Sciences, Department of Health Policy, School of Public Health, Ulaanbaatar, Mongolia; 3 University of Groningen, Faculty of Economics and Business, Groningen, the Netherlands; 4 University of Groningen, Faculty of Science and Engineering, Groningen Research Institute of Pharmacy, the Netherlands; 5 National Institute for Public Health and the Environment (RIVM), Centre for Nutrition, Prevention and Health Services Research, Bilthoven, the Netherlands

## Abstract

**Background:**

Economic evaluations of tobacco control interventions support decisions regarding resource allocation in public health policy. Our systematic review was aimed at identifying potential bias in decision models used to estimate the long-term costs and effects of population-based tobacco control interventions in Asia.

**Methods:**

We included studies conducted in Asian countries and using a modelling technique to evaluate the economic impacts of one or more population-based tobacco interventions in line with the Framework Convention on Tobacco Control (FCTC). We assessed the structure, input parameters, and risk of bias for each model, and performed a narrative synthesis of the included studies.

**Results:**

Nine model-based economic evaluation studies of population-based tobacco interventions were identified. About 60% of the criteria for reporting quality were met in all studies, indicating that reporting generally lacked transparency. The studies were highly heterogeneous in terms of the scope, types, and structures of their models and the quality of input parameters. One-third of the models applied in the studies scored a high risk of bias, with problems mostly falling into the following categories: model type, time horizons, and smoking transition probabilities.

**Conclusions:**

More data are needed to provide high-quality evidence regarding the cost-effectiveness of tobacco control policies in Asia. Strong evidence at the country level hinges on the availability of accurate estimates of the effects of the interventions, the relative risks of smoking, and the price elasticity of the demand for tobacco. Simple transfers of models built in Western populations do not suffice.

**Protocol registration:**

PROSPERO CRD 42019141679.

The Asian continent accounts for the highest production and consumption of tobacco globally [1]. To reduce tobacco use, the World Health Organization proposes a package of interventions, the so-called MPOWER interventions, with proven effectiveness MPOWER includes: **M**onitor tobacco use and prevention, **P**rotect people from smoke, **O**ffer help to quit smoking, **W**arn about the danger, **E**nforce bans, and **R**aise taxes [2,3]. Limited resources for prevention policy may require countries to set priorities [4,5].

Health economic decision models are being increasingly used, as part of health technology assessments (HTAs), within many countries to support priority setting [6]. These models enable tobacco control interventions to be evaluated for their long term consequences. The models support extrapolations from short-term observations and the synthesis of data derived from various sources [7]. The findings of a previous review showed that the majority of the models applied in economic evaluation studies were developed and applied in Western countries [8].

Specific types of bias may occur in model-based economic evaluations. When present, such bias hinders the translation of the economic evaluation results to real life. Previous systematic reviews indicated that all of the examined model-based economic evaluations of smoking cessation interventions had missing information in one or more key domains required for full transferability of these evaluations to a new context [9]. Because these models were often developed in Western countries, assessments of their reporting quality and risk of bias are important prerequisites prior to their application in Asian settings [10].

Hence our review focused on applications in Asia. While previous reviews have examined simulation models used for evaluating tobacco control, they did not consider their application in Asian contexts or examine potential model bias [8]. Therefore, our aim was to conduct a systematic review of the potential bias of decision models used to estimate the costs and effects of tobacco control interventions in an Asian setting. We produced a systematic qualitative synthesis of the studies included in our review.

## METHODS

This systematic review has been registered in the International Prospective Register of Systematic Reviews (PROSPERO) under the following number: CRD42019141679 [11]. We adhered to the Preferred Reporting Items for Systematic Reviews and Meta-Analysis (PRISMA) [12].

### Eligibility criteria

#### Type of population

We reviewed studies conducted in Asian populations, covering a total of 48 countries categorized as belonging to Asia within the WHO country classifications. We excluded Australia and New Zealand.

#### Type of interventions

Primary focus was on the WHO’s ‘MPOWER’ interventions. Studies were included which evaluated a non-clinical, population-based intervention. Individual-oriented interventions, such as cessation support, were excluded from the review, since they can be evaluated by more simple models than population-based interventions.

#### Study design

We reviewed full economic evaluation studies. To be included in the review, studies had to report minimally on intervention costs and health benefits and ideally on all relevant cost consequences and health outcomes. We explored the heterogeneity of model types and structures that are currently being applied within Asian settings, but did require a model-based economic evaluation, that is, use of a mathematical model that simulated both intervention effects and costs.

#### Comparator

There was no restriction on the comparator. The economic evaluation could compare the results of all feasible options in relation to each other and/or to current practices.

### Information sources

A systematic search was performed to identify all relevant studies that satisfied our selection criteria within the following databases: Medline, Embase, Web of Science, and the Cochrane Library. Additionally, we checked the reviews that we identified for further studies.

### Search strategy

In consultation with the medical data specialists, four sets of search strings were used: (1) specific populations/countries classified as Asian, (2) terms related to smoking and tobacco control, (3) combined terms from studies in health economic evaluations, and (4) specific terminology for simulation models. The exact search terms per database are listed in Appendix S1 of the **Online Supplementary Document**. The last search was conducted in November 2019.

### Selection process

After removing duplicates, two authors (AT, YD) independently screened the titles and abstracts followed by the full-text articles. Any disagreements between the two reviewers were resolved through discussions. However, if a disagreement persisted, it was resolved though consultations involving the other authors: GS, EB, GHdB, and TF.

### Data extraction

Data extraction forms were developed using the consolidated health economic evaluation reporting standards (CHEERS) checklists [13]. The two reviewers (AT, YD) independently extracted data using these forms for 30% of the articles included in the review that were randomly selected to ensure consistency. Disagreements were resolved through discussions between the two reviewers (AT, YD), and in cases in which no agreement was reached, a third author (TF) intervened. During this process, data extraction forms were revised and checked by each of the co-authors to ensure consistency in interpretation, and adapt the information extracted from the studies to cover the aspects of relevance to our research question. The final data extraction form used for the current study is shown in Appendix S2 of the **Online Supplementary Document****.**

### Data items

The following data were extracted from each study: (1) overview of study characteristics, (2) model structure, and (3) sources of evidence for model parameters. The data were separately extracted for each intervention type, sub-model, and individual country.

### Risk of bias in individual studies

Quality of reporting in the selected studies was assessed using the 56 items Philips checklist. Items are distributed across three components: structure (n = 20), data (n = 32) and consistency (n = 4) [14]. The percentages of ‘yes’, ‘no’, ‘not applicable’, or ‘unclear’ were calculated for each component. (Appendix S3 of the **Online Supplementary Document**).

Next to this, quality of sources of evidence for model parameters was assessed according to the hierarchy of evidence for economic evaluations [15]. The level of evidence for each model parameter was graded as ‘high’, ‘moderate’, ‘low’, ‘not applicable’ or ‘no source’. A full description of the hierarchy of evidence scales used can be found in Appendix S4 of the **Online Supplementary Document**.

Finally, the bias in economic evaluations (ECOBIAS) checklist was applied to assess the risk of bias [16]. This checklist includes eleven types of bias identified within model-based economic evaluations. Studies were critically assessed for each type of bias, and then ranked as ‘high risk’, ‘moderate risk’, and ‘low risk’ for each item. The average risk of bias was then calculated for each item across all studies. The full checklist is presented in Appendix S5 of the **Online Supplementary Document**.

### Data synthesis

The outcome of this review was a systematic narrative synthesis to present a critical appraisal of the methodological quality and risk of bias of the selected modelling studies. Aim was to assess the suitability of available decision models for the Asian context, using Cochrane guidance [17].

## RESULTS

**Figure 1:**
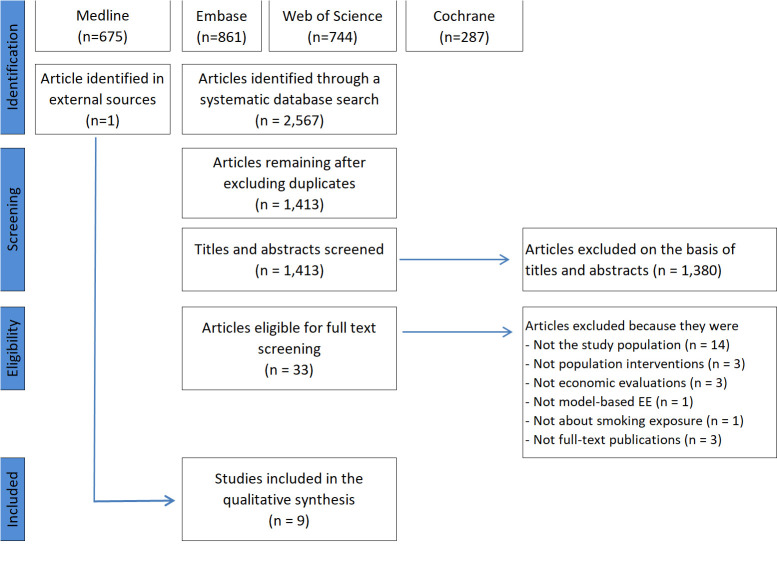
Process of screening and selecting studies for the review.

**Figure 1** presents the details and process relating to the search for and selection of studies for the review. A total of 2567 records were identified during the initial search; nine modelling studies were finally selected for full data extraction.

### Overview of studies included in the review

**Table 1** summarizes the included studies, and Table S2 in the **Online Supplementary Document** adds more details. Most studies (n = 7) focused on interventions conducted within a single country: four studies were conducted in Vietnam,[18, 22, 23, 25] two studies in China, [20,21] and one was in India [24]. Two other studies focused on multiple countries; seven [19] and nine [26] countries respectively. Three studies focused solely on the male population [19-21]. Four studies applied cost-consequence analyses [18,19,25,26] three studies applied cost-effectiveness analyses [22-24] and two studies an extended cost-effectiveness analysis [20,21]. A tobacco tax increase (n = 7) was most often studied. Three studies compared and combined up to four MPOWER population-based tobacco control interventions [20,22,26] namely mass media campaigns, tax increases, labelling of tobacco products, bans on tobacco advertising, graphic warnings, and the promotion of smoke-free work-places.

**Table 1:** Overview of studies included in the review

Author and publication year	Setting; Baseline year	Type of analysis	Intervention	Choice of outcomes		ICER/results	Policy advice/conclusion
**Effects**	**Costs**
Minh et al., 2018 [18]	Vietnam; 2017	Cost- consequence study	Tax increase by 75%–85%	Avoided mortalities	Saved mortality costs	Not presented but health gains and cost savings were found	The government should support efforts to increase the cigarette tax in Vietnam.
Global Tobacco Economics Consortium, 2018 [19]	India, Indonesia, Bangladesh, Philippines, Vietnam, China, Thailand; 2015	Cost-consequence study	One-time increase in the retail price of cigarettes by 50%	Gains in life years	Averted treatment costs	Not presented but health gains and cost savings were found	More health and financial gains for the poorest 20% than for the richest 20% of the population.
					Additional tax revenue		
Verguet et al., 2017 [20]	China; 2015	Extended CEA	Excise tax increased by 75%; smoke-free workplaces	Averted premature deaths	Change in tax revenues; Out-of-pocket payments averted; Poverty prevention	Not presented but health gains and cost savings were found	Significant health and economic benefits for China’s population, especially for the poor.
Verguet et al., 2015 [21]	China; 2011	Extended CEA	One-time tax increase by 50%	Gains in life years	Tax revenue gains; Household expenditure on tobacco; Tobacco-related disease costs; Financial risk protection	Not presented but health gains and cost savings were found	Substantial health and financial benefits for households in China
Higashi et al., 2011 [22]	Vietnam;2006	CEA	Excise tax increased by 55% –85%; Graphic warnings on cigarette packs; Mass media campaigns; Smoking ban in public or work places	DALYs averted	Intervention costs	Median ICER (VND per DALY averted) Taxes increase by 55%–85%: VND 2900;Graphic warnings: VND 500; Mass media campaign: VND 78 300; Ban in public places: VND 67 900; Ban at work: VND 336 800	All interventions were cost-effective. The best options were graphic warnings on cigarette packets and tax increases
Ha et al., 2011 [23]	Vietnam; 2007	CEA	Health education through mass media	DALYs averted	Cost per year	ACER per DALY saved: VND 12 324 059	A mass media campaign on tobacco control was among the most cost-effective interventions
						Very cost-effective (<GDP per capita)	
Donaldson et al., 2011 [24]	India; 2008	CEA	Complete smoking ban	AMI cases averted; Gain in life years	Cost per AMI case averted; Cost per life years gained	Intervention was cost saving	A cost-saving alternative to the current partial legislation in Gujarat
Doran et al., 2010 [25]	Vietnam; 2006	Cost- consequence study	Excise tax increased by 65%-90%	Change in the number of smokers	Total tax revenue	Not presented/less smokers and extra tax revenues	Effective policy option for simultaneously curbing tobacco use and raising revenue
Asaria et al., 2007 [26]	Bangladesh, China, India, Indonesia, Pakistan, Philippines, Russia, Thailand, Vietnam; 2006-2015	Cost- consequence study	Increased taxes on tobacco by 43.2%; A smoke-free workplace; Labelling of tobacco as injurious to health; Ban on tobacco	Deaths averted	Intervention cost per person per year	Not presented	Population-based intervention that could substantially reduce mortality from chronic diseases

GTEC – Global Tobacco Economics Consortium, CEA – cost-effectiveness analysis, VND – Vietnamese Dong, DALY – disability-adjusted life years, ICER – incremental cost-effectiveness ratio, ACER – average cost-effectiveness ratio, GDP – gross domestic products, AMI – acute myocardial infarction

### Model structure

Table S3 in the **Online Supplementary Document** presents a summary of model structures. The most prevalent model type applied was a static simple compartmental model [18-21] that was previously used in international comparison studies [27]. The static models directly link an initial intervention effect in terms of smoking prevalence to total life years gained and costs. The following dynamic models were applied: a state transition model [22]; a dynamic life-table model [23]; and a dynamic population model [25]. Dynamic models estimate health gains over time. Considerable variations existed. For instance, diseases modelled varied from only one [23,24] up to 16 different tobacco-related diseases [26]. All studies performed sensitivity analyses to assess uncertainty associated with key scenarios and parameters. The most commonly used technique was a univariate sensitivity analysis (n = 5).

### Quality of reporting

**Figure 2:**
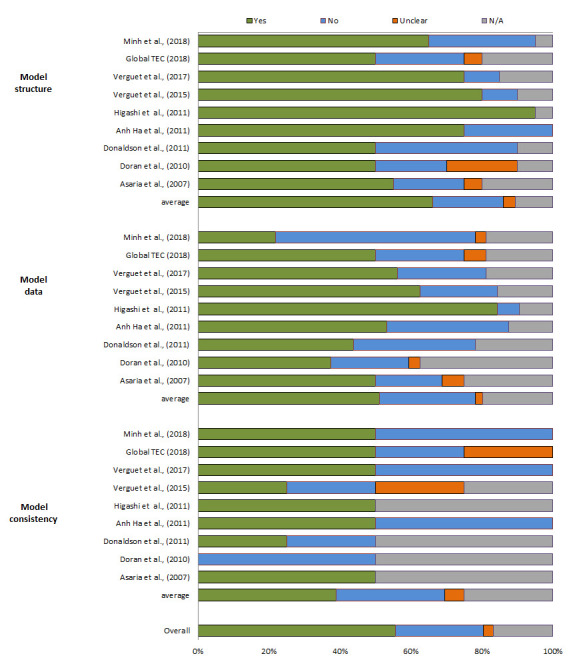
Reporting quality of the studies using the Philips checklist [14].

The results for the studies’ reporting quality are presented in **Figure 2****.** The overall score for the Philips checklist was 56%. The scores for model structure were generally high, with an average of 66%. The score for data was 51% and for consistency 44%. Reporting quality scores per item per study are shown in Appendix Table S4 of the **Online Supplementary Document**.

### Model data

Table S5 in the **Online Supplementary Document** shows sources of evidence and quality of model data for the studies. The quality of disease data in the majority of the studies was assessed to be moderate, as this evidence was obtained from global comparative studies [28,29]. The relative risks of death from tobacco-related diseases were taken from literature (n = 4) and assessed to be moderate quality. The most commonly used data sources were the American Cancer Society’s cancer prevention study (CPS-II) [30] and the WHO’s comparative risk assessment (CRA) [31] that was based on the CPS-II study. Data on intervention effects were assessed to be of moderate quality, either because of the low quality of the study designs in local settings or because studies with high-quality designs were conducted in Western countries. All of the models used high-quality data on smoking prevalence obtained from country-specific surveys. Two studies used disability weights in their models to estimate averted numbers of DALYs [22,23]. Other studies only reported reductions in mortality. High-quality cost data derived from published country-specific findings were used in all studies to estimate unit costs, inpatient costs, and intervention costs.

### Model bias

**Figure 3:**
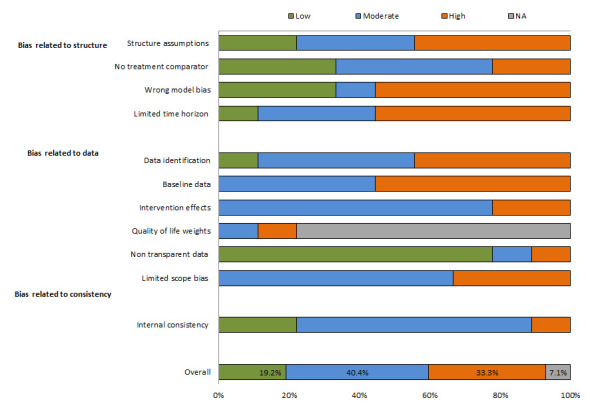
Risk of bias (ECOBIAS) in the reviewed models.

On average, about one-third of the models used entailed a high risk of bias (**Figure 3**). Biases were typically related to quality criteria. More than half of the models did not meet the following quality criteria: selection of appropriate models, a sufficient time horizon for capturing the effects of the interventions, and accurate transition probabilities of the baseline data.

About 40% of the models were found to entail a moderate risk of bias because of their limited scope. At least one of the four uncertainty principles (methodological, structural, heterogeneity, and parameter) were not addressed and the synthesis of data on the effects of interventions was not appropriate. In addition, disability weights (utility data), which are important for estimating the true effects of interventions, were not applied in the majority (78%) of the models. The complete results relating to model bias are shown in Appendix Table S6 in the **Online Supplementary Document**.

## DISCUSSION

Nine studies that used decision models to assess the long-term costs and effects of population-based tobacco interventions in Asia were identified. The studies exhibited a high degree of heterogeneity in terms of how the decision problems were formulated, the scope of the models, and the modelling approaches applied. Our results indicated a considerable room for improving overall levels of transparency in reporting and the quality of the models. The average score for the reporting quality of all studies was 56%. Although poor reporting does not necessary lead to model bias, a lack of transparency undermines assessments of model bias. Notably, we found indications of a high risk of bias in about 33% of the models, while another 40% of the models were found to be associated with a moderate risk of bias.

The model type was a primary cause of model bias. A static compartmental model, entailing the assumption that the introduction of a population-based tobacco intervention would immediately reduce the number of premature deaths, was applied in several studies. This type of model ignores demographic changes and prohibits the use of discount rates for incorporating time preferences within estimates of costs and benefits. Although these static models were associated with rich outcome measures for income distribution relating to additional tax revenues, averted treatment costs, averted out-of-pocket payments, and poverty prevention resulting from tobacco control interventions, they do not yield any insights into the timing or delay of intervention effects and savings.

A second source of bias concerned the model input data, as quality of life effects were ignored in most of the models, with the exception of two studies. One study obtained quality of life weights from a neighbouring country and another reported personal communication as a source without providing a reference. Thus, in most of the studies, the health benefits derived from tobacco control were underestimated as a result of an exclusive focus on life years gained.

Moreover, most of the parameters of the intervention effects were assessed to be of moderate quality, as they were derived from global comparative studies and/or studies conducted in Western countries. By contrast, baseline data on smoking prevalence, obtained from country-specific tobacco surveys, were used in all of the models.

To the best of our knowledge, this is the first Asia-focused review conducted to investigate the potential risk of bias entailed in models used for economic evaluations of tobacco control interventions. In a previous review, existing models applied in tobacco research were categorized by type [8]. In another review conducted in 2017, models used for economic evaluations of smoking cessation interventions were assessed for quality, but not for bias or level of evidence [9]. The study indicated that all of the models lacked one or more key attributes required for full transferability to a new context [9]. Moreover, the majority of the models (58 out of 60) originated in Western countries or in Australia, and only two were applied in Asia. Our review identified nine different models applied in an Asian setting.

Historically, the use of HTA in decision-making in Asia has been slow [32]. The network of Asian HTA agencies, HTAsiaLink, was formally established in 2011, yet not all countries have as of yet joined this initiative [33]. Countries are at different stages to adapt HTA within their unique health care system, and facing different challenges [34]. The efforts are mainly focused on using HTA in support of medicine pricing and reimbursement decisions, rather than population level prevention policy [32,34,35]. This may partly explain why relatively little original modeling efforts could be identified.

In addition, direct transfer of models/study results from Western countries has its limitations, since as indicated in literature Asian countries are in the early stage of the tobacco epidemic.[36] In particularly, this is associated with a relatively low level of the relative risk of death from smoking in Asia, which is a key model parameter in most analyses [37]. Therefore, true intervention effects could be over-estimated when transferred directly from models built for use in Western countries.

A strength of this review was that a comprehensive method for assessing model bias was used, for the three key areas of model bias: structure, data, and consistency [16]. Also, our review encompassed a broad set of population-based tobacco control interventions proposed under the WHO’s MPOWER initiative [3].

A limitation of our review concerns the search method. The electronic database searches were restricted to studies authored in English, and we did not search the grey literature. Apart from practical reasons, the advantage of this restriction might be that the identified studies could be more likely to follow reporting guidelines and have a comparable level of information. However we cannot exclude having missed some good quality studies being published in a non-English journal, or in grey literature. Furthermore, we limited our review to population-based tobacco control interventions, which have been endorsed by the WHO as being effective and efficient, and require more complex modeling than individual-based interventions.

Our studies span the time frame from 2007 to 2018, which has seen increasing attention for model quality. Our checklists were published in 2004 and 2005 and hence reflect standards that were available to all studies reviewed. The models in our review, however, show improvement over time, mostly so since three recent studies [19-21] all used a well-established compartment model developed by the Asian development bank [38].

It is noteworthy that some studies attempted not only to assess the impacts of the investigated interventions on health and total costs but they also examined the contributions of these interventions to the prevention of poverty and the avoidance of catastrophic health expenditure. Economic modelling thus served to inform other national goals, including universal health coverage, the UN sustainable development goals and the WHO-FCTC objectives [39,40].

To satisfy quality standards, future model-based EE studies on tobacco control in Asia could use preferably a dynamic model which tracks the population over a longer time-horizon, and presents properly discounted net present values as outcomes. To include all health benefits, next to mortality, impact on chronic smoking related diseases should be included in the model. Preferably it should allow to analyse the dynamics of smoking cessation and initiation and how these respond to policy by explicitly modeling changes in tobacco use behavior (eg, initiation, cessation and relapse), depending on local data availability. Where possible, the key model parameters need to be based on country-specific data or else on locally relevant sources. Finally, transparent reporting practice following commonly used guidelines for the reporting of economic evaluation studies could minimize the risk of bias in model-based EE studies in Asia [13,14].

Smoking will remain a major public health problem in most Asian countries over the coming decades [37]. Therefore, in line with global initiatives, Asian countries should attempt to implement population-based interventions to end the tobacco epidemic in this region. Towards this goal, countries may develop economic models to evaluate public health policy as part of their HTA initiatives, especially in resource-limited settings where large-scale experiments are not feasible [41]. Clearly, an appropriate methodology and the availability of reliable local data as well as guidance on how to link existing local data to international additional data in an effective manner are prerequisites of high-quality modelling studies [6].

## CONCLUSION

Model-based economic evaluations are an efficient way of informing policy makers and supporting their decisions regarding the best mix of interventions at population-level. However, this requires the availability of high-quality models. Currently, many studies in Asia do not meet this standard and consequently do not attain their goal of adequately supporting decision making. While newer models perform better than less recent ones, much can be gained. Next to this, more local empirical studies would improve the availability of model input parameters. In addition, model developers should pay attention to the structure of their models and ensure the consistency of evidence used to obtain reliable outcomes.

## Additional material

Online Supplementary Document
