# Supplementary material for: Informing policy makers on the efficiency of population level tobacco control interventions in Asia: A systematic review of model-based economic evaluations
Source: J Glob Health. 2020 Dec 19;10(2):020437. doi: 10.7189/jogh.10.020437 (PMC7750019; doi:10.7189/jogh.10.020437)
Supplement: Online Supplementary Document [file jogh-10-020437-s001.pdf]

Electronic supplementary material

Journal of Global Health

Informing policy makers on the efficiency of population-level tobacco control policies in Asia: A systematic review of model-based economic evaluations

Corresponding author:

Ariuntuya Tuvdendorj

[1]Department of Epidemiology, University Medical Center Groningen, Hanzeplein 1, PO Box 30001, 9700 RB Groningen, the Netherlands; t.ariunaa@gmail.com

CONTENTS

Appendix: S1 Search term	3
Appendix: S2 Eligibility criteria	4
Appendix: S3 Quality of reporting checklists	5
Appendix: S4 Quality of sources of evidence	7
Appendix:S5 ECOBIAS Checklist for bias in economic evaluation	9
Table S2: Overview of studies included in the review	10
Table S3: Characteristics and structure of the models	13
Table S4: Quality of reporting	15
Table S5: Quality of model data	17
Table S6: Risk of bias in economic evaluation (ECOBIAS)	20
References	22

Appendix: S1 Search terms

PubMed
("Asia"[Mesh] OR "Far East"[Mesh] OR "Thailand"[Mesh] OR asia*[tiab] OR Banglades*[tiab] OR Bhutan*[tiab] OR India*[tiab] OR Indonesia*[tiab] OR Myanmar*[tiab] OR Nepal*[tiab] OR Sri Lanka*[tiab] OR Thai*[tiab] OR Cambodia*[tiab] OR China[tiab] OR chinese[tiab] OR Hong Kong[tiab] OR hongkong[tiab] OR Japan*[tiab] OR Laos[tiab] OR Malaysia*[tiab] OR Mongolia*[tiab] OR Philippin*[tiab] OR Korea*[tiab] OR Singapor*[tiab] OR Viet Nam*[tiab] OR vietnam*[tiab] OR ((low income*[tiab] OR middle income[tiab]) AND countr*[tiab]) OR (low resource[tiab] AND (setting*[tiab] OR countr*[tiab])) OR Maldiv*[tiab] OR Cook island*[tiab] OR Fiji*[tiab] Or Kiribati*[tiab] OR Nauru*[tiab] OR Niue*[tiab] OR Palau*[tiab] OR Guinea*[tiab] OR Samoa*[tiab] OR Tonga*[tiab] OR Tuvalu*[tiab] OR Vanuatu*[tiab])

AND

("Tobacco"[Mesh] OR "Smoking"[Mesh] OR "Tobacco Use"[Mesh] OR "Tobacco Products"[Mesh] OR "Smokers"[Mesh] OR "Smoke-Free Policy"[Mesh] OR "Nicotine"[Mesh] OR "Tobacco Use Disorder"[Mesh] OR "Smoking Prevention"[Mesh] OR "Smoking Cessation"[Mesh] OR "Tobacco Industry"[Mesh] OR tobac*[tiab] OR cigar*[tiab] OR smok*[tiab] OR antismok*[tiab] OR nicotine*[tiab])

AND

("Economics"[Mesh] OR econom*[tiab] OR cost[tiab] OR costs[tiab] OR costl*[tiab] OR pharmacoeconomic[tiab] OR costing[tiab] OR budget[tiab] OR financ*[tiab] OR expenditur*[tiab])

AND

("Models, Economic" [Mesh] OR "Models, Theoretical"[Mesh:NoExp] OR "Decision Support Techniques"[MeSH] OR "Computer Simulation"[Mesh] OR "Markov Chains"[Mesh] OR model*[tiab] OR econometric*[tiab] OR markov[tiab] OR decision tree*[tiab] OR discrete event*[tiab] OR analytic method*[tiab] OR simulat*[tiab])

Appendix: S2 Eligibility criteria

Inclusion criteria for this review

1. Population: study population must be consists of at least one of countries in Asia.<br>o Selected countries<br>o General population aged over 15+<br>Pregnant women, workers, children, second hand smokers are excluded.	YES	NO	Unclear
2. Intervention: population-based national and international tobacco control interventions must be assessed.<br>o At least one population-based intervention must be assessed.<br>o Protect people from tobacco smoke<br>o Warn about the danger of tobacco<br>o Enforce bans on tobacco advertising, promotion<br>o Raises taxes on tobacco<br>Face-to face intervention: cessation treatments, counsels, nicotine replacements treatments, quit line are excluded.	YES	NO	Unclear
3. Study design: full economic evaluation study must be conducted.<br>o Cost-effectiveness analysis<br>o Cost benefit analysis<br>o Cost utility analysis<br>o Cost minimization analysis<br>Observational, trail-based economic evaluation study, qualitative and case studies, randomized controlled trials, experimental studies, validation studies, adverse drug event studies, (systematic) reviews, editorials, letters, dissertations, books, commentaries and meeting abstracts are excluded.	YES	NO	Unclear
4. Analytic approach: model-based economic evaluation must be applied.<br>o Markov model<br>o Decision tree<br>o Monte Carlo<br>o Dynamic/static<br>Trial-based economic evaluations are excluded.	YES	NO	Unclear
5. Exposure:<br>o Active smoking exposure<br>Second hand smoking exposure, air pollution will be excluded.	YES	NO	Unclear
6. Research article must be:<br>A. In English<br>B. Peer-reviewed<br>C. A full-text publication	YES	NO	Unclear

Appendix: S3 Quality of reporting checklists

The full checklist is provided [1].

#	Questions to consider	Decision: Yes/No/ Unclear/ Not applicable
**Structure**		
S1	Is there a clear statement of the decision problem?	
S1	Is the objective of the evaluation and model specified and consistent with the stated decision problem?	
S1	Is the primary decision-maker specified?	
S2	Is the perspective of the model stated clearly?	
S2	Are the model inputs consistent with the stated perspective?	
S2	Has the scope of the model been stated and justified?	
S2	Are the outcomes of the model consistent with the perspective, scope and overall objective of the model?	
S3	Is the structure of the model consistent with a coherent theory of the health condition under evaluation?	
S3	Are the sources of data used to develop the structure of the model specified?	
S3	Are the causal relationships described by the model structure justified appropriately?	
S4	Are the structural assumptions transparent and justified?	
S4	Are the structural assumptions reasonable given the overall objective, perspective and scope of the model?	
S5	Is there a clear definition of the options under evaluation?	
S5	Have all feasible and practical options been evaluated?	
S5	Is there justification for the exclusion of feasible options?	
S6	Is the chosen model type appropriate given the decision problem and specified causal relationships within the model?	
S7	Is the time horizon of the model sufficient to reflect all important differences between options?	
S7	Are the time horizon of the model, the duration of treatment and the duration of treatment effect described and justified?	
S8	Do the disease states (state transition model) or the pathways (decision tree model) reflect the underlying biological process of the disease in question and the impact of interventions?	
S9	Is the cycle length defined and justified in terms of the natural history of disease?	
**Data**		
D1	Are the data identification methods transparent and appropriate given the objectives of the model?	
D1	Where choices have been made between data sources, are these justified appropriately?	
D1	Has particular attention been paid to identifying data for the important parameters in the model?	
D1	Has the quality of the data been assessed appropriately?	
D1	Where expert opinion has been used, are the methods described and justified?	
D2	Is the data modelling methodology based on justifiable statistical and epidemiological techniques?	
D2a	Is the choice of baseline data described and justified?	
D2a	Are transition probabilities calculated appropriately?	
D2a	Has a half-cycle correction been applied to both cost and outcome?	
D2a	If not, has this omission been justified?	

Appendix: S3 (Continued)

#	Questions to consider	Decision: Yes/No/ Unclear/ Not applicable
**Data**		
D2b	If relative treatment effects have been derived from trial data, have they been synthesized using appropriate techniques?	
D2b	Have the methods and assumptions used to extrapolate short term results to final outcomes been documented and justified?	
D2b	Have alternative assumptions been explored through sensitivity analysis?	
D2b	Have assumptions regarding the continuing effect of treatment once treatment is complete been documented and justified?	
D2c	Are the costs incorporated into the model justified?	
D2c	Has the source for all costs been described?	
D2c	Have discount rates been described and justified given the target decision-maker?	
D2d	Are the utilities incorporated into the model appropriate?	
D2d	Is the source for the utility weights referenced?	
D2d	Are the methods of derivation for the utility weights justified?	
D3	Have all data incorporated into the model been described and referenced in sufficient detail?	
D3	Has the use of mutually inconsistent data been justified (i.e. are assumptions and choices appropriate)?	
D3	Is the process of data incorporation transparent?	
D3	If data have been incorporated as distributions, has the choice of distribution for each parameter been described and justified?	
D3	If data have been incorporated as distributions, is it clear that second order uncertainty is reflected?	
D4	Have the four principal types of uncertainty been addressed?	
D4	If not, has the omission of particular forms of uncertainty been justified?	
D4a	Have methodological uncertainties been addressed by running alternative versions of the model with different methodological assumptions?	
D4b	Is there evidence that structural uncertainties have been addressed via sensitivity analysis?	
D4c	Has heterogeneity been dealt with by running the model separately for different subgroups?	
D4d	Are the methods of assessment of parameter uncertainty appropriate?	
D4d	If data are incorporated as point estimates, are the ranges used for sensitivity analysis stated clearly and justified?	
**Consistency**		
C1	Is there evidence that the mathematical logic of the model has been tested thoroughly before use?	
C2	Are any counterintuitive results from the model explained and justified?	
C2	If the model has been calibrated against independent data, have any differences been explained and justified?	
C2	Have the results of the model been compared with those of previous models and any differences in results explained?	

Appendix: S4 Quality of sources of evidence

The full checklist is provided [2].

**Reference**	**Level of quality of evidence used proposed by Cooper "Use of evidence in decision models" *****	**Decision**
Demographic data	Registration -official sources –from same jurisdiction	High
	Census - from same jurisdiction	High
	Revised projection	Moderate
	Recently published evidence (economic evaluation, report, data synthesis)	Moderate
	Unsourced previous evidences	Low
	Expert opinion (author assumption)	Low
Smoking prevalence data	Periodic surveys conducted using the standardized survey methods — for country of interests	High
	Observational studies (Surveys, cross-sectional studies) – same jurisdiction	High
	Recently published previous evidences (economic valuation, report, – same jurisdiction	Moderate
	Recently published observational studies – different jurisdiction	Moderate
	Unsourced data from previous observational studies –	Low
	Expert opinion (assumption, approximation)	Low
Relative risks	Meta-analysis of cohort studies - same jurisdiction.	High
	Single cohort study – same jurisdiction	High
	Meta-analysis of cohort studies –different jurisdiction	Moderate
	Single cohort study – different jurisdiction	Moderate
	Recently published evidence - (economic evaluation, quality evidence, data synthesis studies)	Low
	Expert opinion	Low
Diseases data	**1** Case series or analysis of reliable administrative databases specifically conducted for the study covering patients solely from the jurisdiction of interest	High
	**2** Recent case series or analysis of reliable administrative databases covering patients solely from the jurisdiction of interest	High
	**3** Recent case series or analysis of reliable administrative databases covering patients solely from another jurisdiction	Moderate
	**4** Old case series or analysis of reliable administrative databases.	Moderate
	**5** Estimates from previously published economic analyses: unsourced	Low
	**6** Expert opinion	Low
Costs data	**1** Cost calculations based on reliable databases or data sources conducted for specific study – same jurisdiction	High
	**2** Recently published cost calculations based on reliable databases or data sources – same jurisdiction	High
	**3** Unsourced data from previous economic evaluation – same jurisdiction	Moderate
	**4** Recently published cost calculations based on reliable databases or data sources – different jurisdiction	Moderate
	**5** Unsourced data from previous economic evaluation – different jurisdiction	Low
	**6** Expert opinion	Low

Appendix: S4 (Continued)

Reference	Level of quality of evidence used proposed by Cooper<br>"Use of evidence in decision models" ***	Decision
Intervention effect	**1**+ Meta-analysis of RCTs with direct comparison	High
	**1** Single RCT with direct comparison between comparator therapies	High
	**2+** Meta-analysis of RCTs with direct comparison between comparator therapies, measuring surrogate outcomes	High
	Meta-analysis of placebo-controlled RCTs with similar trial populations, measuring final outcomes for each individual therapy	High
	**2** Single RCT with direct comparison between comparator therapies, measuring surrogate outcomes	High
	Single placebo-controlled RCTs with similar trial populations, measuring final outcomes for each individual therapy	High
	**3+** Meta-analysis of placebo-controlled RCTs with similar trial populations, measuring surrogate outcomes	Moderate
	**3** Single placebo-controlled RCTs with similar trial populations, measuring surrogate outcomes for each individual therapy	Moderate
	**4** Case-control or cohort studies	Moderate
	**5** Non-analytic studies, for example, case reports, case series	Low
	**6** Expert opinion	Low
Utility weights data (DALY, QALY, Life years)	**1** Direct utility assessment for the specific study from a sample:	High
	**1** Indirect utility assessment from specific study from a patient sample with disease(s) of interest: using a tool validated for the patient population	High
	**2** Indirect utility assessment from specific study from a patient sample with disease(s) of interest using tool not validated for the patient population	
	**3** Direct utility assessment from a previous study from a sample either:	
	**3** Indirect utility assessment from previous study from patient sample with disease(s) of interest: using tool validated for the patient population	
	**4** Indirect utility assessment from previous study from patient sample with disease(s) of interest: using tool not validated for the patient population or method of elicitation unknown	Moderate
	**5** Patient preference values obtained from a visual analogue scale	Low
	**6** Delphi panels, expert opinion	Low
Resource use	Prospective data collection or analysis of reliable administrative data	High
	Recently published results of prospective data collection or recent analysis of reliable administrative data: same jurisdiction	High
	Unsourced data from previous economic evaluations: same jurisdiction	Moderate
	Recently published results of prospective data collection or recent analysis of reliable administrative data: different jurisdiction	Moderate
	Data source not known: different jurisdiction	Low
	Expert opinion	Low

S5 ECOBIAS checklist for bias in economic evaluation

The full checklist is provided [3].

#	Type of bias	Issues addressed (question to consider)	Relevant to study Yes/No/Unclear/Not applicable
Bias related to structure			
1	Structural assumptions bias	Is the model structure in line with coherent theory? Do treatment pathways reflect the nature of disease?	
2	No treatment comparator bias	Is there an adequate comparator, i.e. care as usual?	
3	Wrong model bias	Is the model chosen adequate regarding the decision problem?	
4	Limited time horizon bias	Was a lifetime horizon chosen? Were shorter time horizons adequately justified?	
Bias related to data			
5	Bias related to data identification	Are the methods of data identification transparent? Are all choices justified adequately? Do the input parameters come from high quality and well-designed studies?	
6	Bias related to baseline data	Are probabilities, for example, based on natural history data? Is transformation of rates into transition probabilities done accurately?	
7	Bias related to treatment effects	Are relative treatment effects synthesized using appropriate meta analytic techniques? Are extrapolations documented and well justified? Are alternative assumptions explored regarding extrapolation?	
8	Bias related to quality-of-life weights (utilities)	Are the utilities incorporated appropriate for the specific decision problem?	
9	Non-transparent data incorporation bias	Is the process of data incorporation transparent? Are all data and their sources described in detail?	
10	Limited scope bias	Have the four principles of uncertainty (methodological, structural, heterogeneity, parameter) been considered?	
Bias related to consistency			
11	Bias related to internal consistency	Has internal consistency in terms of mathematical logic been evaluated?	

Table S2 Overview of studies included in the review

Author, Publication year	Target population Setting Baseline year	Study design; Perspective	Comparator Intervention	Choice of outcomes		ICER/results	Policy advice/ conclusion
				Effects	Costs		
Minh et al., 2018[4]	Vietnam 2017<br><br>General population	Cost consequence study;<br><br>Not reported	NA<br><br>Cigarette taxes increase by: 75%- 85%	Number of mortality	Saved mortality cost.	Price increased to 5.7%<br>SADs=63,339;<br>Costs=577 million U$<br>Price increased to 10.5%<br>SADs=116,678<br>Costs=1063 million U$<br>Price increased to 20.9%<br>SADs=232,244<br>Costs=2117 million U$<br>Price increased to 52.3%<br>SADs=581,165<br>Costs=5296 million U$	Increasing the cigarette tax could reduce the substantial health impact of tobacco use, and further result in significant financial savings across society.
GTEC., 2018 [5]	India<br>Indonesia<br>Bangladesh<br>Philippines<br>Vietnam<br>China<br>Thailand<br>2015<br><br>Male smokers	Cost-consequence study ;<br><br>Not reported	Without intervention<br><br>One-time 50% increase in the retail price of cigarettes	Life year gains	Averted treatment costs<br><br>Additional tax revenue	Total life year gained (in million):<br>India: 44.7; Indonesia: 56.8<br>Bangladesh: 17.2; Philippines:14.7<br>Vietnam: 14.3; China: 241;<br>Thailand: 13 Disease cost averted (adjusted for $ PPP, in million)<br>India: 3488; Indonesia: 13350<br>Bangladesh: 507; Philippines: 1964<br>Vietnam: 919; China: 114180<br>Thailand: 2575<br>Additional tax revenues (adjusted for $ PPP, in billion)<br>India: 10.4; Indonesia: 16.4;<br>Bangladesh: 2.6; Philippines: 1.5;<br>Vietnam: 2.4; China: 66.3;<br>Thailand: 3.6	Higher prices of cigarettes provide more health and financial gains to the poorest 20% than to the richest 20% of the population.<br><br>Higher excise taxes support the targets of the sustainable development goals on non-communicable diseases and poverty, and provide financial protection against illness.
Verguet et al., 2017 [6]	China 2015<br><br>Male population	Extended cost-effectiveness analysis;<br><br>Consumer perspective	Without intervention<br><br>Int 1: Excise tax increase: retail price of cigarettes by 75%<br><br>Int 2: Smoke-free workplaces	Averted premature deaths	Change in tax revenue; Averted out of pocket payment Prevented poverty cases; Prevented catastrophic expenditure	Int 1: Avert 24 million premature deaths; Additional US$ 47 billion revenues gains ; Prevent 9 million poverty case; Averted OOP US55$ billion; Prevented 16 million cases of catastrophic expenditure<br>Int 2:Avert 12 million premature deaths; Decrease tax revenue by US$ 7 billion; Prevent 4 million poverty cases	Increased excise taxes on tobacco products and workplace smoking bans can procure large health and economic benefits to the Chinese population, especially among the poor.

Table S2: (Continued)

Author, Publication year	Target population Setting Baseline year	Study design; Perspective	Comparator Intervention	Choice of outcomes		ICER/results	Policy advice/ conclusion
				Effects	Costs		
Verguet et al., 2015 [7]	China 2011<br><br>Male population	Extended cost-effectiveness analysis;<br><br>Consumer perspective	No price increase<br><br>One time tax increase by 50%	Life year gains	Tax revenue gains; Household expenditure on tobacco; Tobacco-related diseas costs; Financial risk protection	Averted mean (95% UR)<br>Years of life gained:<br>231 million (194-268);<br>Additional tax revenues:<br>$703 billion (616-781)<br>Total expenditure on tobacco:<br>$376 billion (232-505)<br>Decreased tobacco-related disease cost: $24 billion (17-26)<br>Financial protection:<br>$1.8 billion (1.2-2.3)	Increased tobacco taxation can be a pro-poor policy instrument that brings substantial health and financial benefits to households in China.
Higashi et al., 2011[8]	Vietnam 2006<br><br>General population	CEA;<br><br>Government	Status quo scenario<br>Int 1: Excise tax increase from 55% to 85%;<br>Int 2: Graphic warning labels on cigarettes pack;<br>Int 3: Mass media campaigns;<br>Int 4: Smoking ban in public (work) place	DALYs averted	Intervention costs	ICER median (95% UI):<br>(VND per DALY averted)<br>Graphic ban: 500 (300- 1200)<br>Taxes : 55%-85%<br>2900(1100-6700)<br>Taxes:55%-75%<br>4200(1700-9900)<br>Taxes: 55%-65%<br>8600(3400-20100)<br>Smoking ban public:<br>67900 (28200- 33200)<br>Mass media campaign:<br>78300 (43700-176300)<br>Smoking ban work:<br>336800 (169300-822900)	All values were negative ICERs, which indicate that the interventions are all cost saving. The government may initially consider graphic warning labels and tax increase, followed by other interventions.
Ha et al., 2011 [9]	Vietnam 2007<br><br>General population	CEA;<br><br>Societal	Without intervention<br>Health education through the mass media	DALYs averted	Cost per year	Costs per year: VND 89 billions<br>DALYs averted per year: 7250<br>ACER per DALY saved:<br>VND 12 324 059<br>Very cost-effective<br>(< GDP per capita)	Health education program to reduce salt intake and a combined mass media on salt, tobacco and cholesterol are the most cost-effective interventions and should purchased first.

Table S2: (Continued)

Author, Publication year	Target population Setting Baseline year	Study design; Perspective	Comparator Intervention	Choice of outcomes		ICER/results	Policy advice/ conclusion
				Effects	Costs		
Donaldson et al., 2011[10]	Gujarat general population (aged > 20)<br><br>India<br>2008	CEA;<br><br>Societal	Partial smoking ban<br><br>Complete smoking ban	cases averted Life years saved	Cost per LY gains	Base case (optimistic-worst);<br>Avert AMI cases:<br>17,478 (53, 361-13,109);<br>Life year gains:<br>437,589(89,1945-45,268)<br>ICER per life year gained w/out medical treatment: US$ 9.13 (2.24-112) ; Cost per AMI case averted: US$229 (37-387)	Implementing a complete smoking ban would be a cost saving alternative to the current partial legislation in terms of reducing tobacco-attributable disease in Gujarat.
Doran et al., 2010 [11]	General population<br><br>Vietnam, 2006	Cost consequence study;<br><br>Government	Business as usual<br><br>Excise taxes level modeled to 65%, 75% and 90%	Change in number of smokers	Total taxes revenue including excise tax revenue and VAT	Number of smokers:<br>12.3 million in 2006 to 13.9 million in 2016 Total taxes revenue: NPV, USD billion; Base case=USD 5.97<br>Excise tax rate= 55%<br>US$10.35-US$10.95<br>Excise tax rate=75%<br>US$10.42- US$11.69<br>Excise tax rate=90%<br>US$10.33- US$12.76	Taxation increases are an effective policy option that can be used by Vietnam government to simultaneously curb tobacco use and raise revenue.
Asaria et al., 2007 [12]	Bangladesh<br>China<br>India<br>Indonesia<br>Pakistan<br>Philippines<br>Russia<br>Thailand<br>Vietnam;<br><br>2006-2015	Cost consequence study	Reference population = SIR method<br>Int 1: Increased taxes on tobacco;<br>Int 2: smoke-free workplace;<br>Int 3: Labelling of tobacco;<br>Int 4: Ban on tobacco	Death averted	Intervention cost per person per year	Death averted: China: 4.5 million; India: 3.1 million; Combined cost of smoking interventions for cost per person per year: Bangladesh: 0.11USD; China: 0.14USD<br>India: 0.16 USD;<br>Indonesia: 0.12USD; Pakistan:0.23 USD; Philippines: 0.13USD; Russia:0.49USD;<br>Thailand:017USD;Vietnam : 0.11USD	Population-based intervention strategies could be substantially reduces mortality from chronic diseases, and makes a major (and affordable) contribution towards achievement of the global goal to prevent and control chronic diseases.

SAD=smoking attributable death

Table S3: Characteristics and structure of the models

Author, publication year	Model name<br>Reference<br>Model type	Model assumption	Smoking categories<br><br>Transition rates	Smoking-related diseases	Relative risk of smoking	Sub-analysis	Time horizon	Discount rate<br>Effects<br>Costs	Sensitivity analysis
Minh et al., 2018[4]	ADB framework<br>[13]<br>Static model	Average PE= 0.25.<br>Initiation PE=0.15 (0.65- 0.15)<br>Mortality rates: 30% to 50%<br>The cost per death: US$9560.8	Current smokers<br>Never smokers<br>Quit rate<br><br>Initiation rate	All-cause mortality	NA	Age group	NA	NA	USA<br>Quit rate and mortality attributable to smoking
GTEC., 2018 [5]	ADB framework<br>[6] [14] [13]<br>Simple static model	Average PE -0.4 (-0.2 to -0.6) from HIC to LMIC<br>PE was twice as large in young PE=-1.27 (15-24)<br>PE=-0.24 (25+)<br>Half of current smokers and future smokers will die<br>Smokers lose on average 10 years<br>Risk reduction by age across income groups	Current smoker<br><br>TR=NA	COPD, Stroke, health disease, lung cancer<br><br>(deaths)	NA	Age group; income quintile	CS	NA	USA<br><br>Price elasticity
Verguet et al., 2017 [6]	Based on #4. Stated that validated model and referred to previous study.<br>Simple static model	Int 1: Price elasticity was -0.38. It was twice as large in younger smokers (15-24 and older) ;<br>Int 2: One-time reduction in smoking prevalence by 9%	Current smokers<br><br>TR=NA	COPD, Stroke, Heart disease, Neoplasm (deaths)	RR for premature mortality by age at quitting	Age group; Income quintile	CS	NA	USA<br>Price elasticity<br>Brand swithing<br>Change in prevalence<br>Poverty threshold
Verguet et al., 2015 [7]	ADB framework<br>[13]<br>Simple static model	Average PE was -0.38<br>(-0.64 to -0.12).<br>Die at age of 71. No additional smoking initiation in > 15 years<br>Half of current and future smokers will die. PE was twice as large in population aged 15-24; No current smokers will would quit in absence of policy 50% mortality rate with no policy.	Current smoker<br><br>TR=NA	COPD, Stroke, Heart disease, Neoplasm<br><br>(deaths)	RR for premature mortality by age at quitting	Age group; Income quintile	50 years	NA	MSA<br><br>price elasticity + treatment cost<br>USA<br>price elasticity

NA: not applied; ADB: Asian development model; PE: price elasticity; TR:transition rate; CS:cohort simulation

Table S3: (Continued)

Author, publication year	Model name Reference Model type	Model assumption	Smoking categories<br><br>Transition rates	Smoking-related diseases	Relative risk of smoking	Sub-analysis	Time horizon	Discount rate<br>Effects Costs	Sensitivity analysis
Higashi et al., 2011[8]	Prevalence model [15] Epidemiological model developed by authors. CostIT model [16]<br><br>Markov model	Int 1: PE of demand was resembled as smoking uptake and smoking participation;<br>Int 2: smoking uptake was assumed to be half the effect on cessation<br>Int 3-4: Intervention effect last for 5 years.	Never smoker, current smoker, former smoker Initiation rate Cessation rate	IHD<br>CVA Lung cancer Oesophagus, Pancreas, bladder, COPD	RR for current smokers	Gender	10 years	3%	USA<br><br>Costs
Ha et al., 2011 [9]	PopMod [17]<br>CostIT model [16]<br>PopMod model	Int: Smoking prevalence will decrease by 1.5% (0.8-2.3%)	Current smoking TR=NA	CVD (incidence)	RR for CVD	Gender	Life table	3%	Best and worst case scenario PSA
Donaldson et al., 2011[10]	Outcome model[18]<br>WHO-CHOICE [12]<br><br>Decision analytic model	Smoke free public places would reduce smoking prev. by 86% - 3.4% Partial ban would reduce SHS by 22%, no change on adult smoking prevalence. Smoke free legislation reduces the prevalence among current adult smokers immediately after implementation by motivating smokers to quit.	Current smoker TR=NA	Acute myocardial infarction incidence	RR for AMI incidence	Gender	10 years	3%	Best and worst case scenario
Doran et al., 2010 [11]	Not reported<br><br>Dynamic population model	10% increase in the final price of cigarette results in a 13.9% switch from cigarettes to rustic tobacco<br>Retail and factory prices are remain constant in real terms<br>% of illegal cigarette sales remain constant	Current smoking Uptake rate Cessation rate	NA	NA	Age Gender,	10 years	3%	USA
Asaria et al., 2007 [12]	SIR: smoking impact ratio WHO-CHOICE<br><br>Static model	Price elasticity -0.4 and -1.2<br>(50% of effect on smoking reduction in smoking prevalence)<br>Proportional reduction in smoking prevalence to all categories of smokers.	Current smokers Former smokers	16 chronic disease<br><br>(mortality)	RR for death	Age, Gender, Country	10 years	NA	Best and worst case scenario

NA: not applied; ADB: Asian development model; PE: price elasticity; TR:transition rate; CS:cohort simulation

Table S4 Quality of reporting

#	Quality items	Minh et al. (2018)	Global tobacco economics consortium. (2018)	Verguet et al. (2017)	Verguet et al. (2015)	Higashi et al. (2011)	Anh Ha et al. (2011)	Donaldson et al. (2011)	Doran et al (2010)	Asaria et al. (2007)
**Structure**										
S1	Is there a clear statement of the decision problem?	✓	✓	✓	✓	✓	✓	✓	✓	✓
S1	Objective consistent with the stated decision problem?	✓	✓	✓	✓	✓	✓	✓	✓	✓
S1	Is the primary decision-maker specified?	✓	✗	✓	✓	✓	✓	✓	✓	✗
S2	Is the perspective of the model stated clearly?	✗	✗	✓	✓	✓	✓	✓	✓	✗
S2	Are the model inputs consistent with the stated perspective?	✗	NA	✓	✓	✓	✓	✗	!	NA
S2	Has the scope of the model been stated and justified?	✓	✗	✓	✓	✓	✓	✓	✓	✗
S2	Outcomes consistent with the perspective, scope, overall objective?	✓	!	✓	✓	✓	✓	✓	✓	!
S3	Is the structure of the model consistent with a coherent theory of the health condition	✓	✓	✓	✓	✓	✓	✗	!	✓
S3	Are the sources of data used to develop the structure of the model specified?	✓	✓	✓	✓	✓	✓	✗	✗	✓
S3	Are the causal relationships described by the model structure justified appropriately?	✓	✓	✓	✓	✓	✗	✗	✗	✓
S4	Are the structural assumptions transparent and justified?	✓	✓	✓	✓	✓	✓	✗	✓	✓
S4	Are the structural assumptions reasonable given the objective, perspective and scop	✓	NA	✗	✗	✓	✓	✗	✓	NA
S5	Is there a clear definition of the options under evaluation?	✓	✓	✓	✓	✓	✓	✓	✓	✓
S5	Have all feasible and practical options been evaluated?	✓	✓	✓	✓	✓	✗	✓	!	✗
S5	Is there justification for the exclusion of feasible options?	✗	NA	NA	NA	NA	✗	NA	✗	✓
S6	Is the chosen model type appropriate given the decision problem?	✗	✓	✓	✓	✓	✓	✗	!	✓
S7	Is the time horizon of the model sufficient to reflect all important options?	✗	✗	NA	✓	✓	✓	✓	✓	✓
S7	Are the time horizon of the model, the duration of treatment described and justified?	✓	✓	✓	✓	✓	✓	✓	✗	✓
S8	Do the disease states/pathways reflect the the disease and interventions?	NA	NA	NA	NA	✓	✗	NA	NA	NA
S9	Is the cycle length defined and justified in terms of the natural history of disease?	✗	✗	✗	✗	✓	✗	✗	NA	NA
**Data**										
D1	Are the data identification methods transparent and appropriate ?	✗	✓	✓	✓	✓	✓	✓	✓	✓
D1	Where choices have been made between data sources, are these justified?	✗	!	✓	✓	✓	NA	✓	!	✓
D1	Has particular attention been paid to identifying data for the important parameters ?	✓	✓	✓	✓	✓	✗	✓	✓	✓
D1	Has the quality of the data been assessed appropriately?	✗	✓	✓	✓	✓	✗	✗	✗	!
D1	Where expert opinion has been used, are the methods described and justified?	NA	NA	NA	NA	NA	✓	NA	NA	NA

Table S4: (Continued)

#	Quality items	Minh et al. (2018)	Global tobacco	Verguet et al. (2017)	Verguet et al. (2015)	Higashi et al. (2011)	Anh Ha et al. (2011)	Donaldson et al. (2011)	Doran et al (2010)	Asaria et al. (2007)
D2	Is the data modelling methodology based on justifiable techniques?	✗	✓	✓	✓	✓	✓	✓	NA	✓
D2a	Is the choice of baseline data described and justified?	✓	✓	✓	✓	✓	✓	✓	✓	✓
D2a	Are transition probabilities calculated appropriately?	!	NA	NA	NA	✓	✗	NA	NA	NA
D2a	Has a half-cycle correction been applied to both cost and outcome?	✗	✗ 1	✗	✗	✗	✗	✗	✗	✗
D2a	If not, has this omission been justified?	✗	NA	✗	✗	✗	✗	✗	✗	NA
D2b	If relative treatment effects have been derived from trial data, have they been synthes	NA	✓	✓	✓	✓	✓	✗	✓	✓
D2b	Have the methods and assumptions used to extrapolate short term results to final out	✗	✗	✗	✗	✓	✓	✓	✗	NA
D2b	Have alternative assumptions been explored through sensitivity analysis?	✗	✓	✓	✓	✓	✓	✓	✓	✓
D2b	Have assumptions regarding the continuing effect of treatment once treatment is comp	✗	✗	✗	✗	NA	✗	✗	✓	✗
D2c	Are the costs incorporated into the model justified?	✓	✓	✓	✓	✓	✓	✓	NA	✓
D2c	Has the source for all costs been described?	✓	✓	✓	✓	✓	✓	✓	NA	✓
D2c	Have discount rates been described and justified given the target decision-maker?	✗	✗	✗	✗	✓	✓	✓	✓	NA
D2d	Are the utilities incorporated into the model appropriate?	NA	NA	NA	NA	✓	NA	NA	NA	NA
D2d	Is the source for the utility weights referenced?	NA	NA	NA	NA	✓	NA	NA	NA	NA
D2d	Are the methods of derivation for the utility weights justified?	NA	NA	NA	NA	✓	NA	NA	NA	NA
D3	Have all data incorporated into the model been described andreferenced in sufficient	✗	✓	✓	✓	✓	✓	✓	✗	✓
D3	Has the use of mutually inconsistent data been justified (i.e. are assumptions and choi	✗	!	✓	✓	✓	✗	✗	NA	!
D3	Is the process of data incorporation transparent?	✓	✓	✓	✓	✓	✓	✓	✗	✓
D3	If data have been incorporated as distributions, has the choice of distribution for each	✗	✓	✓	✓	✓	✓	NA	NA	✓
D3	If data have been incorporated as distributions, is it clear that second order uncertain		✓	NA	✓	✓	✓	NA	NA	✓
D4	Have the four principal types of uncertainty been addressed?	✗	✗	✗	✗	✓	✗	✗	✓	✗
D4	If not, has the omission of particular forms of uncertainty been justified?	✗	✗	✗	✓	NA	✗	✗	NA	✗
D4a	Have methodological uncertainties been addressed by running alternative versions of	✗	✗	✗	✗	✓	✓	✗ 1	✓	✗
D4b	Is there evidence that structural uncertainties have been addressed via sensitivity anal	✓	✗	✓	✓	✓	✗	✗	✓	✗
D4c	Has heterogeneity been dealt with by running the model separately for different subgr	✓	✓	✓	✓	✓	✗	✓	✓	✓
D4d	Are the methods of assessment of parameter uncertainty appropriate?	✗	✓	✓	✓	✓	✓	✗	✓	✓
D4d	If data are incorporated as point estimates, are the ranges used for sensitivity analysis	✗	✓	✓	✓	✓	✓	✓	✗	✓
**Consistency**										
C1	Is there evidence that the mathematical logic of the model has been tested thoroughly	✓	✓	✓	✓	✓	✓	✗	✗	✓
C2	Are any counterintuitive results from the model explained and justified?	✗	!	✗	!	NA	✗	NA	NA	NA
C2	If it has been calibrated against independent data, have any differences been explaine	✗	✓	✗	NA	NA	✗	NA	NA	NA
C2	Have the results of the model been compared with those of previous models and any	✓	✗	✓	✗	✓	✓	✓	✗	✓

Table S5: Quality of model data

Data components	Author, Year of publication	Name of sources	Quality	References
Demographic data	Minh et al., 2018[4]	UN projection	Moderate	[19]
	GTEC., 2018 [5]	UN revision	Moderate	[20]
	Verguet et al., 2017 [6]	UN revision	Moderate	[19]
	Verguet et al., 2015 [7]	UN data & assumption	Moderate	[19]
	Higashi et al., 2011[8]	VINE project	High	[21] [22]
	Ha et al., 2011 [9]	UN Population survey	Low	No source
	Donaldson et al., 2011[10]	Census	High	[23]
	Doran et al., 2010 [11]	VLSS survey	Low	No source
	Asaria et al., 2007 [12]	World bank statistics	Moderate	[24]
Disease data	Minh et al., 2018[4]	Global assumption	Low	No source
	GTEC., 2018 [5]	GBD study	Moderate	[25]
	Verguet et al., 2017 [6]	GBD study	Moderate	[26]
	Verguet et al., 2015 [7]	GBD study	Moderate	[26]
	Higashi et al., 2011[8]	VINE project	High	[22]
	Ha et al., 2011 [9]	Personal communication	Low	No source
	Donaldson et al., 2011[10]	Previous unpublished	Low	No source
	Doran et al., 2010 [11]		N/A	-
	Asaria et al., 2007 [12]	Global projection	Moderate	[27]
Relative risk to disease mortality data	Minh et al., 2018[4]		NA	
	GTEC., 2018 [5]		NA	
	Verguet et al., 2017 [6]		NA	-
	Verguet et al., 2015 [7]		NA	-
	Higashi et al., 2011[8]	CRA, CPS-II	Moderate	[28-31]
	Ha et al., 2011 [9]	CRA	Moderate	[28]
	Donaldson et al., 2011[10]	CPS-II	Moderate	[32]
	Doran et al., 2010 [11]		NA	-
	Asaria et al., 2007 [12]	CPS-II	Moderate	[29][30] [33]
Smoking prevalence data	Minh et al., 2018[4]	GATS survey, GYTS survey	High	[34][35]
	GTEC., 2018 [5]	GATS survey	High	[36-41]
	Verguet et al., 2017 [6]	GATS, China report	High	[42]
	Verguet et al., 2015 [7]	GATS China report; ADB report	High	[42][43]
	Higashi et al., 2011[8]	VLSS and VHLSS	High	[44- 46]
	Ha et al., 2011 [9]	Vietnam National survey	High	[47]
	Donaldson et al., 2011[10]	GATS India Survey	High	[48]
	Doran et al., 2010 [11]	VLSS	High	[44]
	Asaria et al., 2007 [12]	CPS-II (American cancer cohort)	High	[49][50]

Table S5: (Continued)

Data components	Author, Year of publication	Name of sources	Quality	References
Smoking transition rates	Minh et al., 2018[4]		Low	Author assumption
	GTEC., 2018 [5]		NA	
	Verguet et al., 2017 [6]		NA	
	Verguet et al., 2015 [7]		NA	
	Higashi et al., 2011[8]	VLSS and VHLSS	High	[44- 46]
	Ha et al., 2011 [9]		NA	
	Donaldson et al., 2011[10]		NA	
	Doran et al., 2010 [11]	VLSS	High	[44]
	Asaria et al., 2007 [12]		NA	
Intervention effect data	Minh et al., 2018[4]	Local simulation study	Moderate	[51]
	GTEC., 2018 [5]	IARC review	High	[52]
	India	Survey used in India	Moderate	[53]
	Indonesia	Empirical study in Indonesia	Moderate	[54]
	Bangladesh	Survey in Bangladesh	Moderate	[55]
	Philippines	Previous publication	Moderate	[56]
	Vietnam	Cross sectional survey	Moderate	[57]
	China	Previous local publication	Moderate	[58]
	Thailand	Empirical study in Thailand	Moderate	[59]
	Verguet et al., 2017 [6]	Previous publication	Moderate	[14]
		Simsmoke China	Moderate	[60]
	Verguet et al., 2015 [7]	Previous local publication	Moderate	[58] [61]
	Higashi et al., 2011[8]	VLSS survey	Moderate	[62]
		SimSmoke Vietnam	Moderate	[63]
		Case control in Norway	Moderate	[64]
		Cross-section in USA	Low	[65]
		Case control in USA	Moderate	[66]
	Donaldson et al., 2011[10]	Review in USA, AUS, Canada	Moderate	[67] [68]
		Survey in Korea, = base case SimSmoke in USA = SA	Moderate	[69][70]
	Ha et al., 2011 [9]	Review in USA/ Simsmoke in Vietnam	Moderate	[71][63]
	Doran et al., 2010 [11]	Local evidence	High	[72][62]
	Asaria et al., 2007 [12]	Review in global	High	[73]
		Review in USA, AUS, Canada	Moderate	[67]

Table S5:(Continued)

Data components	Author, Year of publication	Name of sources	Quality	References
Utility data	Minh et al., 2018[4]		NA	
	GTEC., 2018 [5]		NA	
	Verguet et al., 2017 [6]		NA	
	Verguet et al., 2015 [7]		NA	
	Higashi et al., 2011[8]	Thailand BOD	Moderate	[74]
	Ha et al., 2011 [9]	Vietnam Study	High	
	Donaldson et al., 2011[10]		NA	
	Doran et al., 2010 [11]		NA	
	Asaria et al., 2007 [12]		NA	
Costing data	Minh et al., 2018[4]		High	[75]
	GTEC., 2018 [5]	Local evidence	High	[41][76-79]
	Verguet et al., 2017 [6]	Local evidence	High	[80-87]
	Verguet et al., 2015 [7]	Local evidence	High	
	Higashi et al., 2011[8]	Local evidence	High	[88]
	Ha et al., 2011 [9]	Local evidence<br>International guide<br>UN estimation in Vietnam	High	[89]<br>[90]<br>[91]
	Donaldson et al., 2011[10]	Local survey	High	[92]
	Doran et al., 2010 [11]		NA	
	Asaria et al., 2007 [12]	WHO-Choice database	to predict country-specific unit costs	[93][94]
Resource use data	Minh et al., 2018[4]		NA	
	GTEC., 2018 [5]		NA	
	Verguet et al., 2017 [6]		NA	
	Verguet et al., 2015 [7]		NA	
	Higashi et al., 2011[8]	CostIt WHO-CHOICE	Moderate	[93]
	Ha et al., 2011 [9]	CostIt WHO-CHOICE	Low	No reference
	Donaldson et al., 2011[10]	WHO-CHOICE model	Moderate	[93]
	Doran et al., 2010 [11]		NA	
	Asaria et al., 2007 [12]	CostIt WHO-CHOICE	Moderate	[93][94]

Table S6 Risk of bias in Economic Evaluation (ECOBIAS)

#	Type of bias	Minh et al., 2018[4]	GTEC. 2018 [5]	Verguet et al., 2017 [6]	Verguet et al., 2015 [7]	Higashi et al., 2011[8]	Ha et al., 2011 [9]	Donaldson et al., 2011[10]	Doran et al., 2010 [11]	Asaria et al., 2007 [12]
Bias related to structure										
1	Structure assumptions	High	High	High	High	Low	Moderate	Moderate	Low	Moderate
2	No treatment comparator	Moderate	Moderate	Moderate	Moderate	Low	Low	High	Low	High
3	Wrong model bias	High	High	High	High	Low	Moderate	Low	Low	High
4	Limited time horizon	High	High	High	Low	Moderate	High	Moderate	Moderate	High
Bias related to data										
5	Data identification	High	High	Moderate	Low	Moderate	Moderate	Moderate	High	High
6	Baseline data	Moderate	Moderate	High	High	Moderate	High	High	Moderate	High
7	Intervention effects	Moderate	Moderate	Moderate	Moderate	Moderate	Moderate	High	High	Moderate
8	Quality of life weights	NA	NA	NA	NA	Moderate	High	NA	NA	NA
9	Non transparent data	Low	Low	Low	Low	Low	Moderate	Low	High	Low
10	Limited scope bias	High	High	Moderate	Moderate	Moderate	Moderate	Moderate	Moderate	High
Bias related to consistency										
11	Internal consistency	Moderate	Low	Moderate	Moderate	Low	Moderate	Moderate	High	Moderate

REFERENCES

[1] "Philips, Z., Ginnelly, L., Sculpher, M., Claxton, K., Golder, S., Riemsma, R., Woolacott, N. and Glanville, J., 2004. Review of guidelines for good practice in decision-analytic modelling in health technology assessment. In NIHR Health Technology Assessme."

[2] S. A. Cooper N, Coyle D, Abrams K, Mugford M, "Use of evidence in decision models: an appraisal of health technology assessments in the UK since 1997. Journal of Health Services Research and Policy 2005; 10: 245-250," 2005.

[3] C. C. Adarkwah, P. F. van Gils, M. Hiligsmann, and S. M. A. A. Evers, "Risk of bias in model-based economic evaluations: the ECOBIAS checklist," *Expert Rev. Pharmacoecon. Outcomes Res.*, vol. 16, no. 4, pp. 513–523, Jul. 2016.

[4] H. Minh, N. Duyen, T. Ngan, … N. N.-… of T. and, and undefined 2018, "Potential health impacts of increasing the cigarette tax in Viet Nam," *ingentaconnect.com*.

[5] "Global Tobacco Economics Consortium. (2018). The health, poverty, and financial consequences of a cigarette price increase among 500 million male smokers in 13 middle income countries: compartmental model study. bmj, 361, k1162."

[6] S. Verguet, G. Tarr, C. Gauvreau, … S. M.-J. of global, and undefined 2017, "Distributional benefits of tobacco tax and smoke–free workplaces in China: A modeling study," *ncbi.nlm.nih.gov*.

[7] S. Verguet, C. Gauvreau, S. Mishra, … M. M.-T. L. G., and undefined 2015, "The consequences of tobacco tax on household health and finances in rich and poor smokers in China: an extended cost-effectiveness analysis," *Elsevier*.

[8] H. Higashi *et al.*, *Cost Effectiveness of Tobacco Control Policies in Vietnam The Case of Population-Level Interventions*, vol. 9, no. 3. Springer International Publishing, 2011, pp. 183–196.

[9] "Ha, Duc Anh, and Dan Chisholm. 'Cost-effectiveness analysis of interventions to prevent cardiovascular disease in Vietnam.' Health policy and planning 26, no. 3 (2010): 210-222."

[10] E. A. Donaldson, H. R. Waters, M. Arora, B. Varghese, P. Dave, and B. Modi, "A Cost-Effectiveness Analysis of India's 2008 Prohibition of Smoking in Public Places in Gujarat," *Int. J. Environ. Res. Public Heal.*, vol. 8, pp. 1271–1286, 2011.

[11] C. M. Doran, J. M. Byrnes, H. Higashi, and K. Truong, "Revenue implications to the Vietnamese government of using taxes to curb cigarette smoking," *Addict. Behav.*, vol. 35, no. 12, pp. 1089–1093, Dec. 2010.

[12] P. Asaria, D. Chisholm, C. Mathers, M. Ezzati, and R. Beaglehole, "Chronic disease prevention: health effects and financial costs of strategies to reduce salt intake and control tobacco use," *Lancet*, vol. 370, no. 9604, pp. 2044–2053, Dec. 2007.

[13] P. Jha, R. Joseph, D. Li, C. Gauvreau, and I. Anderson, "Tobacco Taxes: A Win-win Measure for Fiscal Space and Health. November 2012," *Asian Dev. Bank*, 2012.

[14] S. Verguet, C. Gauvreau, S. Mishra, M. M.-… L. G. Health, and U. 2015, "The consequences of tobacco tax on household health and finances in rich and poor smokers in China: an extended cost-effectiveness analysis," *Elsevier*, 2015.

[15] C. E. Gartner, J. J. Barendregt, W. D. Hall, and E. Gartner, "Predicting the future prevalence of cigarette smoking in Australia: how low can we go and by when?," 2009.

[16] T. T.-T. Edejer and World Health Organization., *Making choices in health : WHO guide to cost-effectiveness analysis*. World Health Organization, 2003.

[17] C. J. L. Murray, D. B. Evans, A. Acharya, and R. M. P. M. Baltussen, "Development of WHO guidelines on generalized cost-effectiveness analysis," *Health Econ.*, vol. 9, no. 3, pp. 235–251, Apr. 2000.

[18] "Richiardi, L; Vizzini, L; Merletti, F; Barone-Adesi, F. Cardiovascular benefits of smoking regulations: The effect of decreased exposure to passive smoking. Prev. Med 2009, 48, 167–172."

[19] "United Nations Population Division Department of Economic and Social Affairs, World Population Prospects: The 2012 Revision," 2013.

[20] "World population prospects: the 2015 Revision New York, NY: United Nations, Department of Economic and Social Affairs, Population Division 2015 [Available from: https://esa.un.org/unpd/wpp/] (Accessed June 15, 2017)."

[21] "The Central Data Processing Centre. Data and results from the 3% sample of the population and housing census 1/4/1999. Hanoi: The Central Data Processing Centre," 2000.

[22] "Vietnam Evidence for Health Policy (VINE) project. Developing the evidence base for health policy in Vietnam. Brisbane and Hanoi: School of Population Health," 2006.

[23] "Government of Gujarat. Census, 2001. Available online: http://www.censusindia.gov.in."

[24] "World Bank World Development Indicators, 2007, World Bank, Washington DC, USA (2007) http://go.worldbank.org/3JU2HA60D0 (accessed Aug 18, 2007)."

[25] "Institute for Health Metrics and Evaluation (IHME). GBD Compare data visualization. Vol. 2017. Seattle, WA: IHME, University of Washington; 2016. [Available from: http://vizhub.healthdata.org/gbd-compare] (Accessed May 20, 2017)."

[26] "Global Burden of Disease Study 2010. Results by risk factor 1990–2010, Institute for Health Metrics and Evaluation, Seattle (2012)."

[27] "CD Mathers, D Loncar Projections of global mortality and burden of disease from 2002 to 2030 PLoS Med, 3 (2006), p. e442."

[28] "Ezzati M, Lopez AD, Rodgers A, et al., editors. Comparative quantification of health risks. Geneva: World Health Organization; 2004."

[29] M. Ezzati, S. J. Henley, M. J. Thun, and A. D. Lopez, "Role of Smoking in Global and Regional Cardiovascular Mortality," *Circulation*, vol. 112, no. 4, pp. 489–497, Jul. 2005.

[30] M. Ezzati, S. J. Henley, A. D. Lopez, and M. J. Thun, "Role of smoking in global and regional cancer epidemiology: Current patterns and data needs," *Int. J. Cancer*, vol. 116, no. 6, pp. 963–971, 2005.

[31] M. Thun, L. Apicella, S. H.- Jama, and undefined 2000, "Smoking vs other risk factors as the cause of smoking-attributable deaths: confounding in the courtroom," *jamanetwork.com*.

[32] "US Department of Health and Human Services. The Health Consequences of Involuntary Exposure to Tobacco Smoke: A Report of the Surgeon General; US Department of Health and Human Services, Centers for Disease Control and Prevention, Coordinating Center for ."

[33] "K Steenland, M Thun, C Lally, C Heath Environmental tobacco smoke and coronary heart disease in the American Cancer Society CPS-II cohort Circulation, 94 (1996), pp. 622-628."

[34] "Viet Nam Ministry of Health. Viet Nam Global Adults Tobacco Use Survey 2015. Hanoi, Viet Nam: MOH, 2016."

[35] "Viet Nam Ministry of Health. Viet Nam Global Youth Tobacco Use Survey 2014. Hanoi, Viet Nam: MOH, 2015."

[36] "International Institute for Population Sciences. Global Adult Tobacco Survey: India report. Mumbai, India. Indian Ministry of Health and Family Welfare. 2010."

[37] "Indonesia Global Adult Tobacco Survey 2011. Ministry of Health, CDC Foundation, U.S. Centre for Disease Control and Prevention, World Health Organization. Jakarta. 2012."

[38] "Ministry of Health and Family Welfare. Global Adult Tobacco Survey: Bangladesh report. World Health Organization, 2009."

[39] “Food and Nutrition Research Institute. 8th National Nutrition Survey: report (Philippines). Taguig City. 2014.”

[40] “Ministry of Health Viet Nam, Hanoi Medical University, General Statistics Office of Vietnam, U.S. Centre for Disease Control and Prevention, World Health Organization. Global Adult Tobacco Survey Viet Nam-2010. 2010.”

[41] “Verguet S, Gauvreau CL, Mishra S, Maclennan M, Murphy SM, Brouwer ED, et al. The consequences of tobacco tax on household health and finances in rich and poor smokers in China: an extended cost-effectiveness analysis. Lancet Glob Heal 2014;3(4):14–8.”

[42] “Global Adult Tobacco Survey (GATS) China Report 2010.”

[43] “Asian Development Bank Tobacco taxes: a win-win measure for fiscal space and health, Asian Development Bank, Manila (2012).”

[44] “General Statistics OfficeVietnam living standard survey (VLSS), 1992–1993 Statistical Publishing House, Hanoi (1994).”

[45] “General Statistics Office. Vietnam living standard survey (VLSS), 1997–1998. Hanoi: General Statistics Office, 2000.”

[46] G. S. Office and General Statistics Office., “Result of the Vietnam household living standards survey 2006. Hanoi: General Statistics Office,” 2007.

[47] “Vietnam National Health Survey 2001/02.”

[48] “International Institute for Population Sciences (IIPS). Global Adult Tobacco Survey India (GATS India) 2009–2010; Ministry of Health and Family Welfare, Government of India: New Delhi, India, 2010.”

[49] “R Peto, AD Lopez, J Boreham, et al. Mortality from tobacco in developed countries: Indirect estimation from national vital statistics Lancet, 339 (1992), pp. 1268-1278.”

[50] “M Ezzati, AD Lopez Measuring the accumulated hazards of smoking: global and regional estimates for 2000 Tob Control, 12 (2003), pp. 79-85.”

[51] “World Health Organization & Viet Nam Ministry of Finance. The tobacco tax simulation model. Viet Nam. Geneva, Switzerland: WHO, 2017.”

[52] “International Agency for Research on Cancer (IARC). Effectiveness of tax and price policies for tobacco control: IARC handbook of cancer prevention.”

[53] “John RM. Price elasticity estimates for tobacco products in India. Heal Policy Plan. 2008;23(3):200–9.”

[54] “Adioetomo M, Djutaharta T, Hendratno. Cigarette consumption, taxation, and household income: Indonesia case study. Washington D.C.; 2005. Barber S, Adioetomo M, Ahsan A, Setyonaluri D. Tobacco economics in Indonesia. 2008.”

[55] “Nargis N, Ruthbah UH, Hussain AKMG, Fong GT, Huq I, Ashiquzzaman SM. The price sensitivity of cigarette consumption in Bangladesh: evidence from the International Tobacco Control (ITC) Bangladesh Wave 1 (2009) and Wave 2 (2010) Surveys. Tob Control. 2014;”

[56] “Quimbo SLA, Casorla AA, Miguel-Baquilod M, Medalla FM, Xu X, Chaloupka FJ. The economics of tobacco and tobacco taxation in the Philippines. 2012.”

[57] “Eozenou P, Fishburn B. Price elasticity estimates of cigarette demand in Vietnam. Florence, Italy; 2001.”

[58] “Hu TW, Mao Z, Shi J, Chen W. The role of taxation in tobacco control and its potential economic impact in China. Tob Control 2010;19(1):58–64.”

[59] “Sarntisart I. An economic analysis of tobacco control in Thailand. Health, Nutrition and Population. Washington D.C.: The World Bank; 2003.”

[60] “Levy D, Rodriguez-Buno RL, Hu T-w, Moran AE. The potential effects of tobacco control in China: projections from the China SimSmoke simulation model. BMJ. 2014;348:g1134.”

[61] “Hu T-w, Mao Z, Shi J, Chen W. Tobacco taxation and its potential impact in China. Paris: International Union Against Tuberculosis and Lung Disease, 2008.”

[62] “Van Kinh H, Ross H, Levy D, et al. The effect of imposing a higher, uniform tobacco tax in Vietnam. Health Res Policy Syst 2006 Jun 26; 4 (1): 6.”

[63] “Levy DT, Bales S, Lam NT, et al. The role of public policies in reducing smoking and deaths caused by smoking in Vietnam: results from the Vietnam tobacco policy simulation model, Social Science & Medicine , 2006, vol. 62 (pg. 1819-30).”

[64] “Hafstad A, Aaro LE, Engeland A, et al. Provocative appeals in anti-smoking mass media campaigns targeting adolescents: the accumulated effect of multiple exposures. Health Educ Res 1997 Jun 1; 12 (2): 227-36.”

[65] “Wakefield MA, Chaloupka FJ, Kaufman NJ, et al. Effect of restrictions on smoking at home, at school, and in public places on teenage smoking: cross sectional study. BMJ 2000; 321 (7257): 333-7.”

[66] “Longo DR, Johnson JC, Kruse RL, et al. A prospective investigation of the impact of smoking bans on tobacco cessation and relapse. Tob Control 2001 Sep 1; 10 (3): 267-72.”

[67] “CM Fichtenberg, SA Glantz Effect of smoke-free workplaces on smoking behaviour: systematic review BMJ, 325 (2002), p. 188.”

[68] “Mullally, BJ; Greiner, BA; Allwright, S; Paul, G; Perry, IJ. The effect of the Irish smoke-free workplace legislation on smoking among bar workers. Eur. J. Public Health 2009, 19, 206–211.”

[69] “Levy, DT; Friend, K; Polishchuk, E. Effect of clean indoor air laws on smokers: the clean air module of the SimSmoke computer simulation model. Tob. Control 2001, 10, 345–351.”

[70] “Kim, B. Workplace smoking ban policy and smoking behavior. J. Prev. Med. Public Health 2009, 42, 293–297.”

[71] “Friend K, Levy DT. Reductions in smoking prevalence and cigarette consumption associated with mass-media campaigns, Health Education Research , 2002, vol. 17 (pg. 85-98).”

[72] “R. Laxminarayan, A. Deolalikar Tobacco initiation, cessation and change: Evidence from Vietnam Health Economics, 13 (2004), pp. 1191-1201.”

[73] “P Jha, FJ Chaloupka The economics of global tobacco control BMJ, 321 (2000), pp. 358-361.”

[74] “Bundhamcharoen K, Teerawatananon Y, Vos T, et al. Burden of disease and injuries in Thailand, 1999. Nonthabury: Bureau of Health Policy and Planning, Ministry of Public Health, 2002.”

[75] “Hoang Anh P T, Thu le T, Ross H, Quynh Anh N, Linh B N,Minh N T. Direct and indirect costs of smoking in Viet Nam. Tob Control 2016; 25: 96–100.”

[76] “Sutrisna B, Surtidewi L, Jusuf A, Hudoyo A, Kusmana D, Setianto B, et al. Estimating the annual cost of smokingrelated diseases in Indonesia. Media Med 2009;43(18):247–53.”

[77] “World Health Organization. Impact of tobacco-related illnesses in Bangladesh. 2005.”

[78] “Ulep V, dela Cruz N. Analysis of out-of-pocket expenditures in the Philippines. Philipp J Dev 2013; 1072:93–122.”

[79] “Le T, Nguyen T, Nguyen H, Nguyen N. Inpatient treatment cost of stroke: an analysis in Ho Chi Minh City 115 People’s Hospital, Vietnam. Value Heal 2016;9(7):649.”

[80] “J She, P Yang, Q Hong, C Bai Lung cancer in China: challenges and interventions Chest, 143 (2013), pp. 1117-1126.”

[81] “C Le, S Zhankun, D Jun, Z Keying The economic burden of hypertension in rural south–west China Trop Med Int

Health, 17 (2012), pp. 1544-1551."

[82] "VW Lee, WK Chan, NL Lam, KK Lee Cost of acute myocardial infarction in Hong Kong Dis Manage Health Outcomes, 13 (2005), pp. 281-285."

[83] "JW Wei, EL Heeley, S Jan, et al. Variations and determinants of hospital costs for acute stroke in China PLoS One, 5 (2010), p. e13041."

[84] "Y Ma, Y Liu, H Fu, et al. Evaluation of admission characteristics, hospital length of stay and costs for cerebral infarction in a medium-sized city in China Eur J Neurol, 17 (2010), pp. 1270-1276."

[85] "E Heeley, CS Anderson, Y Huang, et al. Role of health insurance in averting economic hardship in families after acute stroke in China Stroke, 40 (2009), pp. 2149-2156."

[86] "QY He, X Zhou, CM Xie, ZA Liang, P Chen, CG Wu Impact of chronic obstructive pulmonary disease on quality of life and economic burden in Chinese urban areas Zhonghua Jie He He Hu Xi Za Zhi, 32 (2009), pp. 253-257."

[87] "X Zeng, J Karnon, S Wang, B Wu, X Wan, L Peng The cost of treating advanced non-small cell lung cancer: estimates from the Chinese experience PLoS One, 7 (2012), p. e48323."

[88] "Ross H, Trung DV, Phu VX. The costs of smoking in Vietnam: the case of inpatient care. Tob Control 2007; 16(6): 405–9."

[89] "Flessa S, Dung NT. Costing of services of Vietnamese hospitals: identifying costs in one central, two provincial and two district hospitals using a standard methodology, International Journal of Health Planning and Management , 2004, vol. 19 (pg. 63-77)."

[90] "MSH, International Drug Price Indicator Guide , 2007Boston, MAManagement Sciences for Health."

[91] "UN-EUUN–EU Guidelines for Financing of Local Costs in Development Co-operation with Vietnam, 2007UN Agencies, the Embassies of the EU Member States, and the EC Delegation to Vietnam."

[92] "National Sample Survey Organisation, Ministry of Statistics and Programme Implementation. National Sample Survey Round 60 (NSS-R60), India, 2004. Available online: http://mospi.nic.in/ Mospi_New/site/home.aspx."

[93] "Edejer TTT, Baltussen R, Adam T, et al. Making choices in health: WHO guide to cost-effectiveness analysis. Geneva: WHO, 2003."

[94] "B Johns, T Adam, DB Evans Enhancing the comparability of costing methods: cross-country variability in the prices of non-traded inputs to health programmes Cost Eff Resour Alloc, 4 (2006), p. 8."
